# Supplementary material for: The patient, diagnostic, and treatment intervals in adult patients with cancer from high- and lower-income countries: A systematic review and meta-analysis
Source: PLoS Med. 2022 Oct 20;19(10):e1004110. doi: 10.1371/journal.pmed.1004110 (PMC9584443; doi:10.1371/journal.pmed.1004110)
Supplement: S1 Text — (DOCX) [file pmed.1004110.s013.docx]

**Supplementary Text 1: Search strategy**

**MEDLINE (via OVID)**

1. exp Neoplasms/

2. neoplasm*.tw.

3. tumo$r:*.tw.

4. carcinoma*.tw.

5. cancer*.tw.

6. oncolog*.tw.

7. or/1-6

8. patient delay.tw.

9. help-seeking Interval.tw.

10. patient interval.tw.

11. (time to help-seeking or time to help seeking).tw.

12. (help-seeking delay or help seeking delay).tw.

13. (time to first presentation or time to first consultation).tw.

14. (appraisal interval or appraisal delay).tw.

15. (diagnos* interval or diagnos* delay).tw.

16. (provider delay or system delay or referral delay or investigation delay).tw.

17. (system interval or doctor interval or physician interval or referral interval or primary care interval).tw.

18. (treatment interval or treatment delay or pre-treatment interval or pre-treatment delay).tw.

19. (scheduling delay or scheduling interval).tw.

20. (therapeutic delay or therapeutic interval).tw.

21. *"Appointments and Schedules"/

22. or/8-21

23. 7 and 22

24. (animals not humans).tw.

25. 23 not 24

26. limit 25 to yr="2009 -Current"
