# Supplementary material for: The patient, diagnostic, and treatment intervals in adult patients with cancer from high- and lower-income countries: A systematic review and meta-analysis
Source: PLoS Med. 2022 Oct 20;19(10):e1004110. doi: 10.1371/journal.pmed.1004110 (PMC9584443; doi:10.1371/journal.pmed.1004110)
Supplement: S2 Text — (DOCX) [file pmed.1004110.s014.docx]

**S2 Text: Country socio-economic indicators**

**GNI and HDI**

The GNI per capita, in U.S. dollars, is based on the country’s gross domestic product [1]. GNI measures the value of goods and services produced within a country by all resident producers plus product taxes and all earnings from residents who work abroad. We defined lower-income economies as those with a GNI per capita, calculated using the World Bank Atlas method, of $12,695 or less in 2020 GNI (“low” with less than $1,046, “lower-middle” between $1,046 and $4,095, and “upper-middle” between $4,096 and $12,695) and high-income economies as those with a GNI per capita of $12,696 or more [2]. The HDI is a summary measure of average achievement of a country on key dimensions of human development [3]: a long and healthy life (measured as life expectancy at birth), knowledge (measured by expected years of schooling and mean years of schooling) and a decent standard of living (measured by GNI per capita).

The indicators were extracted for each study according to the respective country and year in which data were collected, to represent the country’s development during the time of diagnosis and treatment. When data collection took place over several years or there was no information on an indicator for a particular year, a protocol was established regarding what data should be extracted (e.g., the indicator for the previous year).

- Extract the values for each respective country corresponding to the year of data collection. If the year of data collection was not reported in the article, impute with the year of publication of the article.
- When there are multiple years specified for the data collection period (e.g., 2007-2010), take the average value of the indicator for those years. If there are no data available for all years in question, take the average of the years for which data are available.
- When there is only one year specified for the data collection period but there is no indicator data for this year, extract the data for the first available year looking backwards (e.g., The data collection period is 2007. There is no HDI for this country for 2007 but there is for 2005, so extract for 2005). If looking backwards there will be a much larger “gap” in years than looking forward, take the data for the year looking forward (e.g., The data collection period is 2007. There is no HDI for 2007. Looking backward the first available is for 2000 and looking forward for 2008. So, in this case, take 2008).

**ICP**

The Policy and Planning Index of Cancer Preparedness (ICP) [4], published in 2019, is available for 28 countries and offers a quantitative measure of the quality of policies aimed to control cancer based on multiple indicators such as the existence and comprehensiveness of a national cancer plan, cancer registries, policies regarding tobacco control, lifestyle and diet, and cancer research, among others. It is scored from 0 to 100, where a higher score reflects better cancer preparedness.

References

1. The World Bank. GNI per capita, Atlas method (current US$). . 2021:<https://data.worldbank.org/indicator/NY.GNP.PCAP.CD.> [Accessed: 16-Sep-2021].

2. The World Bank. World Bank Country and Lending Groups. . 2021:<https://datahelpdesk.worldbank.org/knowledgebase/articles/906519-world-bank-country-and-lending-groups.> [Accessed: 16-Nov-2021].

3. United Nations Development Program. Human Development Index (HDI). Human Development Reports. 2021:<http://hdr.undp.org/en/content/human-development-index-hdi.> [Accessed: 16-Nov-2021].

4. The Economist Intelligence Unit and World Cancer Initiative. Cancer preparedness around the world: National readiness for a global epidemic. 2019. <https://worldcancerinitiative.economist.com/pdf/Cancer_preparedness_around_the_world.pdf>

stylefix
