# Supplementary material for: The patient, diagnostic, and treatment intervals in adult patients with cancer from high- and lower-income countries: A systematic review and meta-analysis
Source: PLoS Med. 2022 Oct 20;19(10):e1004110. doi: 10.1371/journal.pmed.1004110 (PMC9584443; doi:10.1371/journal.pmed.1004110)
Supplement: S3 Text — (DOCX) [file pmed.1004110.s015.docx]

**S3 Text: AARHUS STATEMENT CHECKLIST – SHORT**

The checklist was adapted for the review in a team meeting: several items were dropped due to inapplicability (e.g., questions regarding referral intervals), some questions were further clarified, and common criteria were set. The final checklist contained 15 items: 5 applied to all studies and 10 applied as a function of the interval measured or data source used. The answer options for each question were “yes”, “no”, “uncertain”, or “not applicable”. The checklist was completed independently by two reviewers and disagreements were resolved by a third reviewer. One point was awarded for each “yes” response (“no” and “uncertain” received 0 points each) and the proportion of “yes” responses out of the total that applied was calculated as a measure of risk of bias, resulting in a score ranging from 0% to 100%. Studies with <25% were considered high risk and studies with ≥75% low risk, with the rest considered intermediate.

Response options for each dimension:

If the answer is YES: Low risk of bias

If the answer is PARTIALLY: Uncertain

If the answer is NO: High risk of bias

Each YES gives 1 point towards a final Methodological quality score.

|  | **Question** | **Hints:** |
| --- | --- | --- |
| **For all studies:** | 1. Are the beginning and end points of each interval clearly defined? | This must be done for all intervals measured. |
|  | 2. For all time points and intervals described, are there precise, transparent and repeatable definitions? | Must be true for all time points and intervals measured |
|  | 3. Is the healthcare context in which the study is based fully described? | e.g., description of hospitals and their populations, basic description of the usual diagnostic pathway, etc. |
|  | 4. Do the questions on time points and/or intervals clearly derive from stated definitions? | Is there correspondence between the definitions used and what was measured? |
|  | 5. Do researchers acknowledge the need for theoretical validation and/or make reference to the theoretical framework(s) underpinning measurement and analysis of the time points? | e.g., is any of the models (Andersen, Olesen, Arhus statement) mentioned? Is there any other mention of the need for a theoretical model? |
| **For studies that require an estimate of the date of first symptom** | 6. Is there a discussion of the different biases influencing measurement of this time point? |  |
| **For studies that require measurement of a date of first presentation to healthcare** | 7. Do the researchers discuss the complexity of the date of first  presentation? |  |
| **For studies that require measurement of the date of diagnosis** | 8. Do the researchers use an existing hierarchical rationale for the  date of diagnosis measurement? |  |
| **For studies using questionnaires and/or interviews with patients and/or health-care**  **providers** | 9. Has a validated instrument been used? |  |
|  | 10. Is there some discussion of how reliability and validity (trustworthiness) has been established? |  |
|  | 11. Is there discussion of the different biases influencing measurement of the time points, such as how and when the question is asked and who is being asked? |  |
|  | 12. Is the timing of the interview in relation to the date of diagnosis provided? |  |
|  | 13. Is there any triangulation of self-reported data with other data sources such as case notes? |  |
| **For studies using primary case-note/medical record audit:** | 14. Is there a clear and precise description of how case-note/medical record data were used to ascertain time points, and/or with an acknowledgment of limitations of such data? |  |
| **For database analysis:** | 15. Is there a thorough description of the database chosen including sampling coverage and completeness  of information? |  |
