# Supplementary material for: The patient, diagnostic, and treatment intervals in adult patients with cancer from high- and lower-income countries: A systematic review and meta-analysis
Source: PLoS Med. 2022 Oct 20;19(10):e1004110. doi: 10.1371/journal.pmed.1004110 (PMC9584443; doi:10.1371/journal.pmed.1004110)
Supplement: Attachment — Submitted filename: Reviewer comments.docx [file pmed.1004110.s017.docx]

**Requests from the editors:**

1)      Please revise your title according to PLOS Medicine's style. Your title must be nondeclarative and not a question. It should begin with main concept if possible. Please place the study design (for example, “A modelling study”) in the subtitle (i.e., after a colon). Please consider “Patient presentation, diagnostic, and treatment intervals in adult patients with cancer across various country-level socioeconomic indicators ….: A systematic review and meta-analysis”
A: We have revised the title accordingly to: “The patient, diagnostic, and treatment intervals in adult patients with cancer from high and lower-income countries: a systematic review and meta-analysis”

2)      Abstract:
a)      Please report your abstract according to PRISMA for abstracts, following the PLOS Medicine abstract structure (Background, Methods and Findings, Conclusions) <http://www.plosmedicine.org/article/info:doi/10.1371/journal.pmed.1001419> .

i)      Please rename the “Introduction” to “Background.”

A: Revised.

ii)     Please rename the “Methods and Results” to “Methods and findings.”

A: Revised.

b)      Please include the eligibility criteria

A: Revised.

c)      Please outline all the databases you searched

A: Revised.

d)      Please ensure that all numbers presented in the abstract are present and identical to numbers presented in the main manuscript text.

A: Revised.

e)      Please provide both 95% CIs and p values where appropriate.

A: Revised.

f)      In the last sentence of the Abstract Methods and Findings section, please describe the main limitation(s) of the study's methodology.

A: A section on limitations has been included

3)      At this stage, we ask that you include a short, non-technical Author Summary of your research to make findings accessible to a wide audience that includes both scientists and non-scientists. The Author Summary should immediately follow the Abstract in your revised manuscript. This text is subject to editorial change and should be distinct from the scientific abstract. Please see our author guidelines for more information: <https://journals.plos.org/plosmedicine/s/revising-your-manuscript#loc-author-summary>
A: We have included an author summary as requested.

4)      In line with PLOS Medicine’s guidelines, please update your search to the present time and provide the beginning and end dates of your search.

A: The starting and end dates for the search have been provided in the Method section.

5)      Please avoid use of the term “adult cancer patients” and instead refer to “adult patients with cancer.”

A: The expression has been replaced as suggested.

6)      Please evaluate evidence of publication bias.

A: This has been commented upon in the Discussion section (page 22).

7)      Please indicate in the figure captions the meaning of the bars and whiskers in figure 3.

A: This figure has been replaced with a new one.

8)      Figure 2: Please confirm that the appropriate usage rights apply to the use of this map. Please see our guidelines for map images: <https://journals.plos.org/plosmedicine/s/figures#loc-maps>

A: For Figure 2 we use the Natural Earth data set, which is in the public domain and available from <https://www.naturalearthdata.com>.

*Figure 2: “The base layer map is obtained in R via ggplot2::map_data(“world”), which imports the world map from Natural Earth, which is in the public domain and available from* [*https://www.naturalearthdata.com*](https://www.naturalearthdata.com)*, with terms of use available in* [*http://www.naturalearthdata.com/about/terms-of-use/*](http://www.naturalearthdata.com/about/terms-of-use/)*.”*

9)      The terms gender and sex are not interchangeable (as discussed in <http://www.who.int/gender/whatisgender/en/> ); please use the appropriate term.
A: Ok.

10)     References: 
a)      Please ensure there is no space between in-text reference call outs. For example, “…community [8,9].”

A: This has been corrected.

b)      Please ensure that journal name abbreviations consistently match those found in the National Center for Biotechnology Information (NCBI) databases. <https://journals.plos.org/plosmedicine/s/submission-guidelines#loc-references>.

A: This has been corrected.

c)      Please include access dates for all weblinks and ensure that all weblinks are current and accessible, e.g. #2, 25 etc

A: This has been included.

11)     Please remove the “Declaration of interest”, ‘Funding’, and “Data availability statement,” from the end of the main text. In the event of publication, this information will be published as metadata based on your responses to the submission form
A: This has been removed as suggested.

**Comments from the reviewers:**

**Reviewer #1:**

Dear Authors, thank you for the opportunity to review this systematic review and meta-analysis examining patient, diagnostic and treatment intervals in adult cancer patients using socioeconomic indicators. This is an interesting study, and you should be commended for your transparency in the methodology and explicitly descriptive intention. I do have several comments for your consideration. The introduction adequately describes the interval framework used throughout the manuscript; however, there is not sufficient background information indicating the contributors to the length of time of these intervals and how these vary across regions of different SES. The mention of these prolonged intervals in the introduction within the context of the COVID-19 pandemic is misleading as it is not pertinent to the scope of the study. If the intention is to describe the intervals as a baseline pre-COVID, this should be mentioned explicitly.

A: We have worked extensively to implement all the suggested changes. We have added further information to the introduction to fill in the gaps outlined (details below). We also no longer mention anything related to the covid-19 pandemic, so as not to create any confusion that it is directly related to the goals the current research.

Regarding the methodology, the systematic literature search should be updated as it is almost two years old, and recent publication pertaining to diagnostic and treatment intervals should be captured.

A: We have updated the search as suggested until May 2022.

My last overall note relates to the reporting of results. For clarity, when median/pooled median intervals are presented, the lower and upper confidence bounds (i.e., range in days) also need to be reported.

A: We now report the 95% confidence intervals in square brackets alongside all estimations.

I must also apologize, as I was not able to see line numbers with the provided text and was therefore not able to provide specificity in some of my remarks. I tried my best to provide direction and copy specific sentences to the body of my response, making it easier to control+F. I'd like to thank the authors for taking on this important work and for producing such a well written manuscript. With a few changes and spelling corrections, this review will be a meaningful contribution to the PLOS Medicine collection. Please find my notes below. 
A: Thank you for the thoughtful and constructive review of our manuscript.

Abstract: 
-       Please communicate the information sources for the systematic search by explicitly naming the databases and mentioning that grey literature was also reviewed.
A: This has been revised accordingly in the abstract.

-       To improve clarity, consider changing "first presentation" (when describing patient intervals) to "first presentation to health care facility" to align with the definition provided in the body of the text. 
A: This has been revised accordingly to “first presentation to a healthcare professional”.

-       Identify the number of articles captured from each of the SES regions (ex. of the 329, were 100 HIC and 229 LMIC) and report this in the results.

A: This has been included as suggested in the abstract.

-       Lower and upper bounds need to be provided for each of the pooled estimates (ex. 20 days [range 1-34])

A: This has been included as suggested.

-       Further description of the meta-analytic methods is needed in the abstract. Need to identify the measure that is being used to pool—i.e., describe that pooled median intervals were calculated in units of time (days) with their ranges (lower and upper bound).

A: This has been revised accordingly: “Interval duration was recorded in days and study medians were combined in a pooled estimate with 95% confidence intervals (CI).”

-       The last sentence of the "Results" in the abstract needs to be reworked as it is unclear if there were few studies available from low-income countries in general, or if this pertains to prostate cancers only. Identifying the distribution of studies captured based on HIC and LMIC (as previously mentioned) will aid clarity.

A: This has been revised accordingly: “*The majority of data on the diagnostic and treatment intervals came from high-income countries. Across both high- and lower-income countries, the longest diagnostic intervals were observed for hematological (71 days [95% CI 52-85], e.g., myelomas (83 days [47-145])), genitourinary (58 days [50-77], e.g., prostate (85 days [57-112])), and digestive/gastrointestinal (57 days [45-67], e.g., colorectal (63 days [48-78])) cancers. Similarly, the longest treatment intervals were observed for genitourinary (57 days [45-66], e.g., prostate (75 days [61-87])) and gynecological (46 days [38-54], e.g., cervical (69 days [45-108]) cancers*.”

Introduction:
-       The first sentence of the second paragraph requires citations. 
o       ["It is generally expected that longer interval duration is associated with worse cancer outcomes such as stage migration (i.e., later stage at diagnosis), the need for more extensive therapy with higher toxicity, higher recurrence rates, and higher mortality] 
A: Two references have been added as requested.

-       Additional citations, beyond the systematic review already cited, are required for the following sentence. 
o       [Consistent with this, there is evidence to suggest that shorter times to diagnosis are associated with better outcomes in terms of stage at diagnosis and survival for breast, colorectal, head and neck, testicular cancers, and skin melanoma, with less evidence for pancreatic, prostate, and bladder cancers]
A: Further references have been added to support this sentence.

-       The final sentence of the same paragraph needs regional context to tie in the overarching theme of SES. Are the references representative of both high- and low-income countries? And are the transportable to other populations (i.e., does a four-week delay always have a negative impact in all contexts globally?)
A: This has been commented on as suggested:

*“The majority of the evidence on the effects of early cancer diagnosis and treatment on patient outcomes comes from high income countries (6,8,10-15). It is thus not clear to what extent waiting time thresholds established in higher-income contexts (e.g., for referral to specialist care or initiation of treatment) would have similar effects or be equally feasible in lower-income countries (1,17,18). For instance, when it comes to the duration of intervals on the cancer care pathway, previous research indicates that these vary greatly not only depending on the type of cancer diagnosed (19) but also the socio-economic level of the country (20).”*

-       It is not clear to me why COVID-19 is mentioned in the introduction. The research question is important beyond the context of COVID and there is no link between treatment intervals and COVID throughout the manuscript. Consider adding additional rationale (is this to establish a baseline of delays to make meaningful comparisons as the pandemic plays out?) or moving this section to the discussion. 
A: To avoid confusion, we have removed the references to the covid-19 pandemic from the introduction.

The introduction adequately describes the interval framework used throughout the manuscript; however, there is not sufficient background information indicating the contributors to the length of time of these intervals and how these vary across regions of different SES.

A: We have added some information on barriers to cancer care:

*“Common barriers to early diagnosis and care such as poor health literacy, cancer stigma, lack of access to diagnostic tests and treatment services, and financial, geographical, or logistical barriers are likely to be exacerbated in lower-income contexts, contributing to longer intervals on the cancer care pathway and worse patient outcomes (2,20,22). Longer times to diagnosis and treatment and later stage at diagnosis are likely some of the multifactorial patient- and health system-driven causes of the larger cancer burden and lower survivorship in lower-income countries (21).”*

-       A greater emphasis on what this study contributes to the literature is needed. Brand et al. (ref 13) published a SR on a nearly identical topic, with a similar number of articles captured (n=319) in 2019.   
A: The excellent review by Brand and colleagues has been a very useful guide for us, however, there are multiple differences between the two reviews. Brand et al. focused on lower-income countries only (high-income countries were not considered). Their research question was broader (also including barriers) and the number of studies identified reporting on the intervals of interest was actually lower than 319. They also considered childhood cancers and could only pool data on interval duration for childhood and breast cancers (the rest were grouped into “other” due to lack of sufficient studies). We have added the following information to the introduction to clarify how our review is different from previous reviews:

*“Previous reviews have offered information about the duration of different intervals focusing on specific cancers (23-26). Another recent review reported on the duration of different intervals for childhood and breast cancer in lower income countries (20). However, there has been no review that offers an overview of the duration of the different intervals across different cancer sites and comparing high- and lower-income countries. Until recently, there was also no validated methodology for reliably combining median interval duration data using meta-analytic techniques (27).”*

Methods:
-       The search is outdated and needs to be updated. If the authors' goal is to avoid capturing articles reporting on treatment intervals during COVID-19 times, consider adding COVID-19 as a "NOT" term in the search strategy. Restricting based on time will unnecessarily penalize studies published recently that are not related to COVID.

A: We have now updated the search until May 2022, which led to the inclusion of 81 new articles, making a total of 410 articles included in the review. We tested the suggested strategy to exclude article on COVID-19 from the search but eventually decided against it because it may lead to the exclusion of articles that also report on data from before the pandemic. We figured there may be pre-post comparison articles comparing interval duration for periods before vs. after the pandemic and we eventually extracted pre-pandemic data from several such articles (data collected for periods after the covid-19 pandemic was excluded, as per our registered review protocol).

-       Complete the first sentence under the "inclusion criteria" sub-heading.
o       ["Studies reporting data on the length of any of the three intervals of interest for any cancer site in adult cancer patients presenting with primary cancers….were included"]

A: This has been revised accordingly.

-       Mention if authors of studies that were potentially eligible but had missing information were contacted—corresponds to Figure 1, Reports Assessed for Eligibility, with exclusion reason Incomplete outcome reporting. If authors were contacted, up to how many times before considered excluded?

A: We did not contact authors and now mention this in a footnote of the figure.

o       Please correct the spelling error in this section of Figure 1."an interval of interest" rather than "if".

A: This has been corrected.

-       Complete the first sentence under the subheading "exclusion criteria" similar to comment above. 
A: This has been revised accordingly.

-       It is unclear what the purpose of the independent and blind screening of 35% of the abstracts retrieved was for. Please clarify if the intention was to pilot and assess concordance between the two reviewers before continuing. Make this explicit in the text as well. 
A: This has now been explained in more detail:

*“Because we expected to identify a large number of abstracts for screening, to reduce reviewer workload, we planned to perform the screening individually (i.e., that abstracts be screened by one reviewer only), if we could establish that agreement between reviewers was sufficiently high. To assess this, we performed independent and blind screening of 26% of the abstracts by two reviewers.”*

-       Please complete the first sentence under the subheading "data extraction", similar to comments above.

A: This has been revised accordingly.

-       Under the "statistical analysis" subheading, please add the number of studies that reported mean intervals (rather than median intervals) in addition to the percentages provided. 
A: This has been revised accordingly.

*“…this was n=52 (1514%), n=15 (5%), and n=70 (1615%) for the patient, diagnostic, and treatment interval, respectively”*

Results:
-       Consider including the division of articles from HIC's and LMIC's in the first sentence of the results section. (ex. 300 from HICs and 29 from LMICs, or whatever the true breakdown is).

A: This information has now been included:

*“The majority of articles reported data form high-income countries (294 or 72%), with 116 (28%) reporting on lower income countries: 48 (12%) reporting data on upper-middle, 43 (10%) on lower-middle, and 24 (6%) on low-income countries.”*

-       When median intervals are presented, the lower and upper bound must be presented in the body of the text. This comment relates to all sections (abstract, all intervals/results, and the discussion).

A: We now include the 95% CI in square brackets alongside all pooled estimates.

-       In the fourth paragraph under the subheading "Patient Intervals," please quantify what a large number of studies means by reporting the exact number (see below).
o       ["Notable differences supported by a large number of studies included a pooled median of 50 compared to 29 days for breast"]. i.e., is this 50? Report the exact number

-       Under the subheading "diagnostic intervals" please quantify few studies by reporting the exact number.
o       ["Few studies from less developed countries were available…"]

-       Please report the number of studies representing "small" in the sentence noted below.
o       ["72 days, R2=15%, although this difference was much less pronounced and based on only a small number of studies, see Table S7)."]
-       Under the subheading "treatment intervals" please report the number of studies implied by "very small"
o       ["The number of studies from lower-income countries was again very small, so we discuss the results averaged across all countries (Table 3)."]

A: Regarding the previous four comments: The Results section has now been revised and vague references to a “large” or “small” number of studies have either been removed or quantified by specifying the exact number of studies. Also, the number of studies/pooled estimates included in each analysis are reported in Tables 1 to 3.

-       Under the subheading "sensitivity analyses" please quantify what is implied by "in most cases"
o       ["In most cases (for example, could be n=7) intervals remained unchanged or changed up to 7 days."]

A: This has now been quantified as suggested (see “Sensitivity analysis” section), for instance:

*“In most cases intervals remained largely unchanged (i.e., change up to ±1 day for 5, 8, and 9 cancer groups on the patient, diagnostic, and treatment intervals, respectively) or changed up to 6 days (for 3, 3, and 0 cancer groups on the patient, diagnostic, and treatment intervals, respectively).”*


Discussion: 
-       The limitation of the study methods discussed in the second paragraph should be expanded upon and should consider the global context. For example, when grouping leukemias together, CML and ALL to be pooled together, which may have drastically different times to presentation, diagnosis, and treatment. A worked example included in the discussion, like the case of leukemia, would be helpful for all readers and highlight the limitation of this method. 
A: We have now revised this paragraph accordingly (please also note that it has been moved to page 20 to improve text flow):

*“Whereas the grouping of specific cancer sites into general main groups has been useful for descriptive and comparative purposes, it is also limited. The specific cancer sites may present unique challenges and circumstances when it comes to diagnosis and treatment, something that is also reflected in the variation of the pooled intervals within the main cancer groups considered. To take hematological malignancies as an example, the pooled median diagnostic interval for this group was 71 days [52-85]. Desegregating the data further showed very different diagnostic intervals for leukemias (30 days [13-87]) compared to lymphomas (69 days [44-82]) and myelomas (83 days [47-145]). However, even within these more specific groups there could be large variation in the clinical manifestation and diagnostic process depending on the type of cancer. To illustrate, in a study based on the UK’s Hematological Malignancy Research Network, the median duration of the diagnostic interval was 13 days for acute lymphocytic and 10 days for acute myeloid leukemia but 42 days for chronic lymphocytic and 9 days for chronic myeloid leukemia (37). Discussing the unique diagnostic and treatment circumstances of all cancer sites reported is beyond the scope and possibilities of the review, it is, however, something readers should bear in mind when interpreting our results.”*

-       In the fourth paragraph, discussing cancer registries, please consider adjusting the language. Cancer registries do exist in lower-income countries, however the infrastructure to support their effectiveness may not be established. Adding "The expansion and creation of cancer registries…." may be more appropriate to recognize the efforts already in place.
o       ["The creation of cancer registries or large cancer epidemiological databases in lower-income countries as an investment in national cancer control planning is one of the priorities suggested to reduce cancer care disparities worldwide (14)."]
A: This has been modified as suggested.

-       The limitation of sample size, such that few studies were available from LMICs, needs to be highlighted, as it is an important and crucial result in and of itself. 
o       ["There were fewer differences between high and lower-income countries on the diagnostic and treatment intervals at least partially due to lack of enough data."]
-       As a descriptive study, it is important to highlight the implicit result that is the distribution of publications from HICs and LMICs. 
A: To address these comments, we have added the following text to the discussion:

Page 17*: “Importantly, only 28% of the identified articles reported data from lower income countries and mostly on the patient interval.”*

Page 18: *“Fewer studies were available from lower-income countries, especially reporting on the diagnostic and treatment intervals.”*

-       Please correct the spelling from "polled" to "pooled" in the sentence below.
o       ["Our sensitivity analysis showed that the removal of studies with highest potential for bias did not substantially affect polled estimates for the more common cancers"]
A: This has been corrected.


**Reviewer #2**: Alex McConnachie, Statistical Review

Petrova et al have done a systematic review and meta-analysis of studies reporting treatment intervals for (adult) cancer, looking at differences between cancer types and between countries based on national income and the Human Development Index. This review considers the statistical aspects of the paper.

In short, these are very good. The authors use methods derived to combine estimates of medians across studies, which has been shown to work better than previously used methods, based on converting everything to a mean and standard error. My comments are not about the methods, so much as the presentation of the results.

A: Dear Dr. McConnachie, thank you for the helpful suggestions to improve our manuscript. Please note that as per the suggestion of Reviewer 1, the literature search has been updated to include articles published in the past 2 years. Whereas the main conclusions remain unchanged, we have added 81 new articles, and the specific numeric estimates have changed for the majority of cancer types.

The last two paragraphs of the results report some sensitivity analyses, which generally did not affect the results very much, though for the few exceptions, the authors list some cancers for which treatment intervals are reduced. What these estimates reduced to is reported, but not what from, so without going to find the original estimate in the tables, it is not possible to judge these results. For the benefit of the reader, it would help to give the original and sensitivity estimates in the same place.

A: We have now revised this section to include both the original and reduced estimates to facilitate comprehension. For instance:

*“To illustrate, in the case of neurologic/brain cancers the pooled patient interval was reduced with 33 days from 48 days [9-616] to 15 days [7-730], in the case of sarcoma it was reduced with 30 days from 75 days [29-130] to 45 days [25-141], and in the case of melanoma it was reduced with 15 days from 85 days [39-334] to 70 days [25-217].”*

Table 1 gives a lot of information, but does not give confidence intervals for the estimated differences between low- and high-income countries. P-values could also be given.

A: We have now included the estimated differences between studies from high- and lower-income countries and their respective 95% confidence intervals and exact p-values.

Because the *metamedian* package we have used to meta-analyze the data (or any other relevant software we are familiar with) does not provide median-specific meta-regression tests to estimate these differences, the results reported are based on Wilcoxon Rank Sum tests (wilcox.test function in R). Because we have used the “median of medians” method from the *metamedian* package, which provides unweighted pooled estimates, the results from the Wilcoxon Rank Sum tests are generally in strong agreement with the observed differences in the meta-analyses stratified according to country income level. In our opinion, this demonstrates the usefulness of this test on this occasion, given the lack of a specific test within the *metamedian* package. To give an example, in the case of breast cancer, the pooled patient interval for high-income countries is 32 [24-45] days, for lower-income countries it is 58 [35-92] days, and the estimated differences is 25 [7-61] days, p=0.002.

We have explained this in the Analysis section:

*“Second, differences in interval duration (in number of days) between studies conducted in high- and lower-income countries were estimated based on Wilcoxon Rank Sum tests, generating a 95% confidence interval (CI) for the estimated differences.”*

Specific p-values (and estimates of explained variance) for comparisons between high- and lower-income countries are also available for the supplementary meta-regression models conducted in the *metafor* package and reported in Tables S6-S9.

I did not like figure 3. I think forest plots would be better. If low- and high-income estimates are reported in pairs, then the margin of the forest plot could show the estimated difference, with a confidence interval and p-value. Note, I would report the actual p-value, not use stars to indicate significance.
A: We have replaced Figure 3 with a forest plot as suggested.

The supplement also needs a little attention, most notably in terms of the table numbering (the tabs of the spreadsheet do not match the contents).

A: We have revised the table numbering and corrected the errors.

In general, I would encourage the authors to present confidence intervals for estimates of differences (can CIs be produced for interval ratios?), and report actual p-values rather than using a system of asterisks.
A: We have now followed this recommendation throughout the manuscript and in the supplementary files and exact p-values are reported. Regarding interval ratios (reported in the section Relative contribution of intervals and Table S10), we have now also reported 95% CI following Bonett DG, Price Jr RM. Confidence intervals for ratios of means and medians. Journal of Educational and Behavioral Statistics. 2020 Dec;45(6):750-70.

*“To compare and estimate the relative contribution of the different intervals, following previous studies (19), we calculated the ratios between the different intervals. Specifically, because the diagnostic interval was the longest interval for the majority of cancers, following methods by Bonett and Price (36), we calculated the ratios of the diagnostic to the patient interval (DI/PI), the diagnostic to the treatment interval (DI/TI), and the patient to the treatment interval (PI/DI) with their respective 95% CI. This was only done for studies that reported the duration of all three intervals in the same sample of patients and intervals were considered to be significantly different when the 95% CI for their ratio excluded 1.”*


**Reviewer #3:**

A: Thank you for the thoughtful and constructive review of our manuscript. Please note that as per the suggestion of Reviewer 1, the literature search has been updated to include articles published in the past 2 years. Whereas the main conclusions remain unchanged, we have added 81 new articles, and the specific numeric estimates have changed for the majority of cancer types.

Major comment 1. The text reads: ""This was evaluated using a short form of the 'Aarhus checklist' (4) developed to assess the quality of studies that measure intervals on the cancer treatment pathway. The checklist contains questions regarding interval definitions, measurement, use of theoretical frameworks, discussion of validity, biases, and limitations of measurement, among others. The checklist was completed independently by two reviewers and disagreements were resolved by a third reviewer. Studies with scores <25% were considered high risk and studies with ≥75% low risk, with the rest considered intermediate (see Supplementary Text 3)." So, the bias assessment process does not seem to include survivorship bias / patient selection. This is a key issue, as survivors (who are able to be surveyed or interviewed, to provide information about their patient interval from symptom onset to presentation) may have atypical diagnostic intervals to those patients who
die shortly after their diagnosis. see schema Keeble S et al. International Journal of Cancer 2014 <https://pubmed.ncbi.nlm.nih.gov/24515930/>  (which forms part of the citation list), and also Koo M et al, Neoplasia 2017 review paper <https://pubmed.ncbi.nlm.nih.gov/29253839/> where the matter is explained schematically.

A: This is correct, the checklist does not include specific items that assess susceptibility to patient selection/survivorship bias, which would be relevant for a large number of studies. To address this issue, we have now 1) mentioned it as a major limitation in the abstract and 2) provided more detailed discussion about its implications in the Discussion section. Thank you for pointing us to this issue and the specific references.

Abstract: *“This review covered a large number of studies conducted worldwide but is limited by survivor bias and the inherent complexity and many possible biases in the measurement of time points and intervals in the cancer treatment pathway.”*

Discussion, page 21: *“An additional limitation to consider is survivor bias, which is especially relevant for studies using patient interviews and questionnaires. This method of data collection was also especially frequent in studies conducted in lower-income countries that reported on patient intervals. Survivor bias is a type of patient selection bias, where patients dying soon after symptom onset or patients who are too ill to take part in a research study are excluded (47,48). This could result in biased patient-reported estimates of interval duration and limit generalizability, because patients who die shortly after diagnosis or are too ill to participate may have atypical interval duration. Studies using medical records are less prone to selection and recall biases (47), however, they have other limitations (e.g., it is assumed that the information recorded during the consultation is complete and accurate, which may not be the case) (48).”*

Major comment 2. There is no theoretical explanation as to why the review excluded the primary care interval - particularly as several studies do exist that report this. This is likely to be a crucial component of any diagnostic process in most (?all) countries, particularly low-income countries which the review focuses on. I am not sure that these data / studies should be excluded - if they are excluded a strong rationale needs to be provided for their exclusion.

A: We agree that this is a limitation to the scope of the current review, which we have mentioned in the abstract and in the Discussion section:

Abstract (paragraph on limitations): *“In addition, the sub-intervals that compose the diagnostic interval (e.g., primary care interval, referral to diagnosis interval) were not considered.”*

Discussion, page 21: *“Another limitation is that we did not consider the sub-intervals that compose the diagnostic interval (e.g., primary care interval, referral to diagnosis interval) (4). We wanted to make comparisons across countries with very different health systems and we preferred to focus on the more generalizable measure of the total diagnostic interval. Future reviews should consider the sub-intervals that compose the patient (e.g., appraisal vs. help-seeking interval) and diagnostic (e.g., primary care interval vs. referral to diagnosis interval) intervals to offer a more comprehensive understanding of the patient journey to diagnosis in different contexts.”*

We would also like to clarify that studies reporting on the primary care interval were not excluded per se, they were rather not considered for inclusion to begin with, as our study protocol focused only on the three intervals described in the manuscript (patient, diagnostic, and treatment). As a result, the search strategy did not include keywords specific to the primary care interval, so even if we were to extract information from the studies that entered the search, they would only represent a small part of all relevant studies. Finally, just an observation: although we did not keep record of this while reviewing the studies for eligibility, our impression is that the very large majority of articles where we encountered reports of the primary care interval were from high income countries.


More minor points:

1.      Abstract, sentence: "The longest diagnostic intervals were observed for hematological, gynecological, and digestive/gastrointestinal cancers, and the longest treatment intervals for prostate cancer, with few studies available from lower-income countries." It would be more informative if specific cancer sites are called out, as these groups include widely different (with respect to diagnostic timeliness) cancers, eg. myeloma (known to have very long diagnostic intervals) versus leukaemia (know to have very short intervals) are both 'haematological', and similarly ovarian (long intervals) vs endometrial (short intervals) are both 'gynaecological', etc. for other cancers. Please could you state specific cancer sites (as exemplars).

A: We have now included also information about specific cancers in the abstract, in particular, for those with the longest intervals within the group.

*“Across both high- and lower-income countries, the longest diagnostic intervals were observed for hematological (71 days [95% CI 52-85], e.g., myelomas (83 days [47-145])), genitourinary (58 days [50-77], e.g., prostate (85 days [57-112])), and digestive/gastrointestinal (57 days [45-67], e.g., colorectal (63 days [48-78])) cancers. Similarly, the longest treatment intervals were observed for genitourinary (57 days [45-66], e.g., prostate (75 days [61-87])) and gynecological (46 days [38-54], e.g., cervical (69 days [45-108]) cancers.”*

2.      I found all the material deposited in Open Science Framework very useful and excellent practice, well done to authors' team.

A: Thank you.

3.      Where text reads: "…..such as stage migration (i.e., later stage at diagnosis)" - I think this needs rephrasing, e.g. to read "later stage at diagnosis _because of stage migration" (my underline, to be removed).

A: This has been rephrased to “It is generally expected that longer interval duration is associated with worse cancer outcomes such as later stage at diagnosis…”

4.      What does the following mean: "In the case of HDI, the high vs. lower groups were created using k-means clustering." Please explain.

A: We have now provided more information in the Method section:

*“First, stratified meta-analyses were performed for high vs. lower-income countries (high vs. lower GNI and higher vs. lower HDI). In the case of HDI, the higher vs. lower groups were created using k-means clustering (i.e., the groups were based on their “natural” grouping based on k=2 centroids). This method was chosen because of the skewed distribution of the HDI variable, which would result in artificial grouping using other methods such as creating equal-count groups.”*

Essentially, we noticed that the distribution of the HDI was heavily skewed for many cancers (with the majority of countries having high scores). As a result, creating “high” vs. “low” groups based on more conventional methods such as equal-count groups or similar would produce a very artificial division, where countries with relatively high HDI scores are counted towards the “low” group. The method of k-means clustering avoids that by forming what would be two “natural” groups as a function of variable distribution (although this often results in very unbalanced n across groups).

Finally, we would like to clarify that this “high” vs. “low” grouping was used for illustrative purposes only, as the meta-regression analysis used the original continuous scores (mentioned on page 10 in the manuscript).

5.      In the exclusion criteria (online appendices) many papers are excluded because of reporting the 'WRONG OUTCOME'. However, these studies did not report any wrong outcome per se, only an outcome not being considered by the review. Please rephrase.
A: We have changed the wording in Figure 1 and the supplemental materials. Thank you for the thoughtful and constructive review of our manuscript.

**Reviewer #4**: The authors are congratulated for their valuable manuscript that is a meaningful addition to the scientific literature and to cancer policy development. The meta-analysis methodology is robust and results are presented well. Consideration can be made to strengthen the Discussion and expand the data synthesis.

A: Thank you for the thoughtful and constructive review of our manuscript. Please note that as per the suggestion of Reviewer 1, the literature search has been updated to include articles published in the past 2 years. Whereas the main conclusions remain unchanged, we have added 81 new articles, and the specific numeric estimates have changed for the majority of cancer types.

Major considerations:
1. Data synthesis and interpretation: while understanding that these sub-analyses may be outside the scope of the current study, the following considerations can be made given the quality of data:

(a) Information on study design and percent of studies utilizing interviews, medical records or database by country: as governments and international stakeholders evaluate the feasibility of including early diagnosis indicators in national programmes, it would be helpful to present the information from Table S4 into the main text. What percentage of countries obtain this information through databases and/or medical records? Can any comment be made on differences in interval estimates according to data source (ie, are patient interviews reliable)?

A: We have now included more detailed information derived from Table S4 into the Results section:

*“One hundred (24%) articles used a combination of sources to obtain interval data. In particular, 146 (36%) used questionnaires or interviews (with patients or health professionals), 215 (52%) used medical records, and 141 (34%) used large databases (e.g., population-based cancer registry data, the USA National Cancer Database (NCDB), SEER, or similar). Use of questionnaires or interviews was more frequent in studies from lower-income countries (n=77, 66% vs. n=69, 23% of studies from high-income countries), whereas use of large databases was more frequent in studies from high-income countries (n=137, 47% vs. n=4, 3% of studies from lower-income countries). The use of medical records was relatively similar (n=150, 51% for high-income, and n=65, 56% for lower-income countries, respectively).”*

We have desegregated the information as a function of country socio-economic level instead of specific countries due to the very large number of countries represented in the review. Please note that percentages do not sum to 100% because some studies used a combination of methods.

We have also added similar information regarding the intervals reported as a function of country socio-economic indicators:

*“Reporting the patient interval was more common in studies from lower-income countries (n=89, 77% vs. n=88, 30% of studies from high-income countries), whereas reporting the treatment interval was more common in studies from high-income countries (n=194, 66% vs. n=49, 42% of studies in lower-income countries). The percentage of studies reporting the diagnostic interval was similar for high- and lower-income countries with n=86 (29%) vs. n=35 (30%), respectively.”*

Please note that percentages do not sum to 100% because some studies reported on more than one type of interval.

Overall, studies from lower-income countries most often reported patient intervals and used interviews or questionnaires. In contrast, studies from high-income countries most often reported treatment intervals and used large databases.

Regarding the reliability of interval estimates according to data source, we have now included some discussion about this on page 21:

*“An additional limitation to consider is survivor bias, which is especially relevant for studies using patient interviews and questionnaires. This method of data collection was also especially frequent in studies conducted in lower-income countries that reported on patient intervals. Survivor bias is a type of patient selection bias, where patients dying soon after symptom onset or patients who are too ill to take part in a research study are excluded (47,48). This could result in biased patient-reported estimates of interval duration and limit generalizability, because patients who die shortly after diagnosis or are too ill to participate may have atypical interval duration. Studies using medical records are less prone to selection and recall biases (47), however, they have other limitations (e.g., it is assumed that the information recorded during the consultation is complete and accurate, which may not be the case) (48).”*

(b) Total interval: why only a minority of studies evaluate total interval, is there sufficient data to perform a meta-analysis for total intervals for select cancers?

A: Unfortunately, we do not have data on the duration of the total interval. Per protocol we extracted data on the median (or mean) duration of the three specific intervals (patient, diagnostic, and treatment). This means that, for instance, articles that reported on the total interval but not on any of the specific intervals have been excluded. In addition, if a study reported on all three intervals, it does not mean that the total interval was reported. Although the three intervals add up to form the total interval, mathematically, adding up the three medians would not provide us with the correct median for the total interval. For this reason, we could not conduct meta-analysis on the total interval based on the current study protocol and available data.

(c) Variability among high-income countries: the authors could consider a sub-analysis comparing high-income countries. This could include a comparison between high-income countries that have had dedicated public health programmes in early diagnosis (eg, UK). Data on the availability of early diagnosis programmes can be gathered from databases like WHO and/or OECD.

A: Thank you for this suggestion. We searched these and other databases and also consulted with specialists external to our team in an effort to find systematically gathered information on the existence of public health programs for early detection of cancer, available for a number of high-income countries that would permit us to conduct meaningful analysis. Unfortunately, the information we found was mostly about the existence and/or implementation of screening programs, something we considered too out of scope, given that the focus of our review is symptomatic cancers and that screening programs target only specific cancer types and age groups.

Although we could not find specific information about early detection programs such as those in the UK, we did come across a measure that provides data on cancer policies for multiple high-income countries. In particular, the Index of Cancer Preparedness (ICP), and specifically its “Policy and planning” component “offers a quantitative measure of the quality of policies aimed to control cancer based on multiple indicators such as the existence and comprehensiveness of a national cancer plan, cancer registries, policies regarding tobacco control, lifestyle and diet, and cancer research, among others”.

We have now compared the duration of intervals from studies in high-income countries based on their policy and planning scores. The index is not available for all high-income countries included in the review but the countries that represent the largest number of studies (such as USA, UK, Canada, Netherlands, Australia, Spain, …) are included. The results show that higher scores on policy and planning are associated with shorter patient and diagnostic intervals for several cancer locations (i.e., breast, head and neck, and digestive/gastrointestinal, see changes in Method section, Results section and Table S7).

Page 14: *“Considering patient interval variation within high-income countries only, patient intervals were longer in studies conducted in countries with lower scores on the Policy and Planning ICP for breast (34 days [34-52] vs. 7 days [7-7] for countries with higher scores) and head and neck cancer (60 days [31-64] vs. 30 days [21-35] for countries with higher scores, see Table S7).”*

Page 15: *“Considering only high-income countries, diagnostic intervals were longer in studies conducted in countries with lower scores on the ICP Policy and Planning for digestive/gastrointestinal cancers (84 days [64-92] vs. 72 days [67-97] for countries with higher scores, see Table S7).”*

Page 16: *“Considering only high-income countries, treatment intervals were longer in studies conducted in countries with higher scores on the ICP Policy and Planning for genitourinary cancers (83 days [65-107] vs. 57 days [50-60] for countries with lower scores, see Table S7), and specifically for prostate cancer (83 days [77-107] vs. 60 days [57-61] for countries with lower scores).”*

We have now also discussed these results on page 19-20 in the Discussion section.

*“The additional analysis focused on high-income countries included in the Index of Cancer Preparedness (33) revealed that the existence and implementation of diverse cancer-directed policies is related to shorter patient and diagnostic intervals for some cancers. In the case of the patient interval for breast and head and neck cancers and the diagnostic interval for digestive/gastrointestinal cancers, lower Policy and Planning scores on the Index of Cancer Preparedness were associated with longer intervals. These results suggest that in high-income contexts, the implementation of cancer-directed policies such as national cancer plans including strategies for primary prevention and early detection of cancer (46) could have positive effects on diagnostic delays. Whereas it is not clear what policies exactly may be driving these effects and having in mind that these results are at best preliminary, they offer much needed evidence regarding the potential effects of cancer policies on relevant outcomes (46).”*

(d) At risk population: recognizing that this may not have been collected, did the authors identify population groups who were at higher risk for delays? If so, can a comment be made on this?

A: Unfortunately, we did not collect any data regarding the factors associated with longer intervals, as it was not among the goals of our review. However, we have deposited all review materials and study information in an open-access data repository, should other researchers want to use it as a base to explore further research questions like this one. For instance, one could easily locate all studies that reported interval duration for a specific cancer type, and extract further information about the factors associated with longer intervals to answer new research questions.

2. Discussion: 
(a) Diagnostic and treatment requirements for select cancer types: why have the authors referenced that such a discussion would be beyond the scope and possibilities of this manuscript? If data are being synthesized by cancer, a brief comment highlighting differences between select cancers (particularly those most represented in this study) is relevant and valuable.

A: We have now described in more detail the most relevant differences between the pooled intervals for the different cancer types that are considered in the same group throughout the Results section. For example:

Page 15: *“There was significant variation for the specific hematological malignancies with a much shorter pooled diagnostic interval for leukemias (30 days [13-87]) compared to lymphomas (69 days [44-82]) and myelomas (83 days [47-145]). In the case of genitourinary cancers, diagnostic intervals were longest for prostate (85 days [57-112]) and renal cancers (72 days [38-102]). For digestive/gastrointestinal cancers, pooled diagnostic intervals were longer for colorectal cancers (63 days [48-78]) compared to upper gastrointestinal cancers (43 days [30-62]). Within gynecological cancers, the longest diagnostic intervals were observed for cervical cancer (75 days [49-95]).”*

Page 16: *“Within genitourinary cancers, the longest treatment intervals were observed for prostate cancer (75 days [61-87]) and within gynecological cancers for cervical cancer (69 days [45-108]). Within head and neck cancers, treatment intervals for thyroid cancers were notably longer than for the other specific cancer sites (165 days [67-502], compared to pooled estimates between 26-34 days for the rest of the cancer sites). Within digestive/gastrointestinal cancers, treatment intervals were longer for upper gastrointestinal cancers (40 days [22-51]) than for colorectal cancers (18 days [14-22]).”*

We have also added further discussion about this on page 20, by giving hematological malignancies as an example:

*“To take hematological malignancies as an example, the pooled median diagnostic interval for this group was 71 days [52-85]. Desegregating the data further showed very different diagnostic intervals for leukemias (30 days [13-87]) compared to lymphomas (69 days [44-82]) and myelomas (83 days [47-145]). However, even within these more specific groups there could be large variation in the clinical manifestation and diagnostic process depending on the type of cancer. To illustrate, in a study based on the UK’s Hematological Malignancy Research Network, the median duration of the diagnostic interval was 13 days for acute lymphocytic and 10 days for acute myeloid leukemia but 42 days for chronic lymphocytic and 9 days for chronic myeloid leukemia (37). Discussing the unique diagnostic and treatment circumstances of all cancer sites reported is beyond the scope and possibilities of the review, it is, however, something readers should bear in mind when interpreting our results.”*

(b) Screening: the authors state that "another strategy that could contribute to reducing the negative impact of lengthy patient delays in this context is the successful implementation of cancer screening programmes." It should be noted that <10% of cancers are found by screening in HIC; screening programmes are not effective means for early diagnosis, particularly in lower-income countries. Such a statement should be strongly referenced if to be endorsed or can be avoided.

A: For simplicity this statement has been removed.

3. Data visualization: the authors present relevant information on relative contribution through interval ratios. It would be informative to convert this into a figure disaggregated by high-income countries and lower-income countries. This will assist the reader to identify particular bottlenecks. 
A: The interval ratios can only be calculated from the studies that report all three intervals, which were unfortunately too few to segregate by country income level (see Table S10). However, we have now transformed Figure 3 into a forest plot that shows detailed data on the intervals for both high and lower-income countries.

Minor consideration: 
1. Classification scheme: the authors use categorization of the National Cancer Institute (reference 25). Can the authors provide feedback on why ICD-10 was not utilized?

A: We decided to use this classification to accommodate the studies that do not report specific diagnostic details or ICD codes. We did not keep record of this but our impression is that not reporting such information was common in 1) studies from lower income countries and 2) studies on patient intervals (e.g., published in psychology journals).

2. "Burden" versus "survival": on pg 11, the authors state that "…longer diagnostic intervals for breast cancer and longer treatment intervals for gynecological cancers in lower-income countries, both of which could be contributing to the higher burden…"; would consider exchanging "lower survival" for "higher burden" to increase precision. 
A: This has been modified as suggested.

3. Commentary on longer treatment intervals for genitourinary cancers: the authors present a valid hypothesis, but it would be worthwhile to state that this is a hypothesis for the longer treatment interval unless data are available that the more indolent behaviour explains the longer interval. It could also be the result of concerns about treatment morbidity or stigma.

A: We have added an additional sentence to reflect that:

*“Thus, one hypothesis that could be tested in future research, is that the long treatment intervals for prostate cancer are due to many patients undergoing “watchful waiting”, although concerns about treatment morbidity or stigma could also play a role in some contexts.”*

In this context, we have also commented on one result with the newly added Index of Cancer Preparedness:

*“In addition, studies from high-income countries with lower scores on the Index of Cancer Preparedness on Policy and Planning (e.g., Italy, USA, Spain) reported shorter treatment intervals for prostate cancer than studies from countries with higher scores (e.g., Australia, Netherlands, Canada, Germany). This could also be due to higher implementation of watchful waiting for prostate cancer or controlled treatment delays in certain contexts.”*

4. Intervals in Figure 3 (pg 22): all three figures have the title "Patient Interval"; it is probable that the second and third are "Diagnostic" and "Treatment"

A: Thank you, this error has been corrected in the new figure.
