# Supplementary material for: The patient, diagnostic, and treatment intervals in adult patients with cancer from high- and lower-income countries: A systematic review and meta-analysis
Source: PLoS Med. 2022 Oct 20;19(10):e1004110. doi: 10.1371/journal.pmed.1004110 (PMC9584443; doi:10.1371/journal.pmed.1004110)
Supplement: Attachment — Submitted filename: R2 reviewer comments.docx [file pmed.1004110.s018.docx]

Requests from Editors:

1. Please use square brackets for in-text reference call outs, e.g., “… [6,7]."

A: This has been changed as requested.

Comments from Reviewers:

Reviewer #1: Dear Authors,

Thank you for the opportunity to review this revised manuscript describing patient, diagnostic, and treatment intervals in adults with cancer. The authors have made significant revisions and expanded the capture of their search strategy to reflect the most up-to-date literature. All suggestions by reviewers were adequality incorporated and justified within the text and supplement. Please be careful with spelling in some places (ex. in the second paragraph of the results, please correct the following sentence: "The majority of articles reported data form high-income countries" Correct "form" to "from"). With these changes, this systematic review and meta-analysis using novel methods will be one of the largest and most meaningful contributions to the existing literature. 

A: We have now carefully proofread the manuscript. Thank you for the constructive review of our paper.

Reviewer #2: Alex McConnachie, Statistical Review

I thank the authors for their consideration of my original points. I am happy with all of their responses.

One typo: on page 11, the patient to treatment interval is abbreviated as PI/DI, when I assume it should be PI/TI.

A: This has been corrected.

One question: is there a way to apply weights when using a Wilcoxon Rank Sum test to compare groups of studies and generate confidence intervals? I had a quick look online, and couldn't find anything conclusive, but if there is a way to do it, then it might be slightly better.
A: We searched for a way to apply weights to the test, too, but did not find anything that seemed suitable. Thank you for the constructive review of our manuscript.

Reviewer #3: Thank you to the authors for carefully considering and addressing my comments and questions and further improving their manuscript.
A: Thank you for the constructive review of our manuscript.

Any attachments provided with reviews can be seen via the following link:

__________________________________________________
In compliance with data protection regulations, you may request that we remove your personal registration details at any time.  (Use the following URL: <https://www.editorialmanager.com/pmedicine/login.asp?a=r>). Please contact the publication office if you have any questions.
